# A Shallow Convolutional Learning Network for Classification of Cancers Based on Copy Number Variations

**DOI:** 10.3390/s19194207

**Published:** 2019-09-27

**Authors:** Ahmad AlShibli, Hassan Mathkour

**Affiliations:** Department of Computer Science, College of Computer and Information Sciences (CCIS), King Saud University, Riyadh 11543, Saudi Arabia; mathkour@ksu.edu.sa

**Keywords:** CNV, cancer classification, deep learning, convolutional neural network, VGG16

## Abstract

Genomic copy number variations (CNVs) are among the most important structural variations. They are linked to several diseases and cancer types. Cancer is a leading cause of death worldwide. Several studies were conducted to investigate the causes of cancer and its association with genomic changes to enhance its management and improve the treatment opportunities. Classification of cancer types based on the CNVs falls in this category of research. We reviewed the recent, most successful methods that used machine learning algorithms to solve this problem and obtained a dataset that was tested by some of these methods for evaluation and comparison purposes. We propose three deep learning techniques to classify cancer types based on CNVs: a six-layer convolutional net (CNN6), residual six-layer convolutional net (ResCNN6), and transfer learning of pretrained VGG16 net. The results of the experiments performed on the data of six cancer types demonstrated a high accuracy of 86% for ResCNN6 followed by 85% for CNN6 and 77% for VGG16. The results revealed a lower prediction accuracy for one of the classes (uterine corpus endometrial carcinoma (UCEC)). Repeating the experiments after excluding this class reveals improvements in the accuracies: 91% for CNN6 and 92% for Res CNN6. We observed that UCEC and ovarian serous carcinoma (OV) share a considerable subset of their features, which causes a struggle for learning in the classifiers. We repeated the experiment again by balancing the six classes through oversampling of the training dataset and the result was an enhancement in both overall and UCEC classification accuracies.

## 1. Introduction

The human genome sequence is subject to variations (insertions, deletions, or inversions) of different sizes, which range from the single nucleotide base to an entire chromosome [1]. Structural variations are defined as those with lengths that exceed 1000 bases [2]. Copy number variations/alterations (CNVs/CNAs), are considered among the most important structural variations [1,3] because they are located in 12% of the human genomes [4] and because of their correlation with several diseases [5].

Cancer is a category of disease that involves uncontrolled abnormal cell growth and can spread to other tissues [6]. The number of cancer cases is increasing annually, with 9.6 million deaths reported in 2018 [7]; therefore, several studies are attempting to investigate cancer causes and treatment.

Several studies have indicated relationships between CNAs and several cancer types, such as breast cancer [8], lung cancer [9], and colorectal cancer [10]. Detecting CNVs and understanding their associations with cancer can help in early cancer diagnosis and achieve a higher rate of successful treatment [11]. Profiling CNVs can also be used to classify cancer types and distinguish benign from malignant tumors [12].

In the following sections of this paper, a review of the previous work to classify cancers based on CNVs is provided. Section 2 describes the dataset, the process of preparing the data for the experiment, and the architecture of our convolutional network. Section 3 specifies how the data are divided and the experiments are conducted. In Section 4, we present the results and compare the performance of the evaluated models. Section 5 summarizes our conclusions. Finally, we discuss our future work in Section 6.

### 1.1. Background

Computer scientists use different methods to deduce the type of cancer based on the level of CNVs. Li et al. [9] compared the single-nucleotide polymorphism-based CNVs of the entire genome of early stage adenocarcinoma and squamous cell carcinoma samples. They used maximum-relevance-minimum-redundancy (mRMR) [13] to prepare a ranked feature list, and incremental feature selection (IFS) [14] to elect the optimal set of features, which were 266 CNV features in their experiment. This set was then used to discriminate the two tumors. The chosen features were inputted to the nearest neighbor algorithm to classify the samples within the different tumor classes using the jackknife cross-validation method. The study identified eight genes that were the best candidates to distinguish between these two cancers.

Zhang et al. [15] applied the same methods (mRMR and IFS) to select 19 features out of 24,174 features from the dataset of CNVs. The feature selectors calculated the maximum feature relevance to the label and the minimum redundancy to rank all the features. This classification was examined by adding highly ranked features one by one to the chosen set. In total, the dataset contained 3480 samples of six cancer types (breast adenocarcinoma, colon and rectal carcinoma, glioblastoma multiforme, kidney renal clear cell carcinoma, ovarian serous carcinoma (OV), and uterine corpus endometrial carcinoma (UCEC)). As a classifier, they used the Dagging algorithm [16] with 10-fold cross validation to achieve an accuracy of 75%.

Ricatto et al. [17] proposed a tumor classification method using a pipeline that applied a distributed fuzzy discretizer [18] to the training data; subsequently, they used it to learn a distributed fuzzy decision tree [18], and finally produced a fuzzy rule-based classifier. A set of 50 interpretable rules were used to infer kidney tumor types out of three classes, with an accuracy of 93%.

Yuan et al. [19] proposed a 2D convolutional neural network classifier (DeepCNA), which combined CNVs and cell lines of Hi-C data to extract high-level features for tumor classification. Testing the classifier against a dataset of 25 cancer types yielded an accuracy of 57.4%.

Elsadek et al. [20] used a filter-based feature selection algorithm and information gain algorithm to select and rank 16,381 features of six cancer types, and applied six well-known machine learning algorithms (support vector machine, Dagging, random forest, decision trees, neural network, and logistic regression) as classifiers to compare their accuracies among other metrics. The best accuracy was reported at 85.9% using logistic regression.

### 1.2. Copy Number Variation-Based Classification Problem

The classification of cancer types based on CNVs demonstrates the limitation of high dimensionality as each CNV is considered a classification feature. The classification techniques follow different paths to overcome this obstacle: one way is to reduce the number of dimensions by electing the features that express tighter correlations to classes they represent, and then to use only these significant features with a wide range of classification algorithms, hoping that they prove sufficient for a targeted accuracy. This method, if successful, can facilitate further studies on these features to investigate the underlying associations and causations between CNVs and the cancers they indicate. However, this approach assumes individuality in the CNV label indications, and overlooks the possibility of having a group of CNVs networked to act as a single feature, the value of which is composed of a combination of its involved CNVs, and maybe with different weighted contributions. From a mathematical perspective, given the problem (*X*, *Y*), where *X* is the set of all *N* raw features and *Y* is the set of *M* class labels:

$$X\;=\;\left\{x^{1},\;x^{2},\;x^{3},\;\ldots \;,x^{N}\right\},\;Y\;=\;\left\{y^{1},\;y^{2},\;y^{3},\;\ldots \;,\;y^{M}\right\}.$$

The feature selection algorithm determines a subset $X^{\prime }\subset X$ that satisfies:

(1) reduction of the dimension of the problem $\left|X\prime \right|\ll \left|X\right|$
(2) for a training dataset $\left(X_{K},Y_{K}\right)$, the probability $p(Y_{k}|X\prime_{k}):1\leq k\leq K$, has a maximum value.

Alternatively, the classifier produces a set of processed features $X\prime \prime =\left\{x\prime \prime^{1},\;x\prime \prime^{2},\;x\prime \prime^{\prime 3},\;\ldots \;,x\prime \prime^{U}\right\}$, in which each feature $x\prime \prime^{u}\;=\;g\left(X\right):1\leq u\leq U$, which results from processing all the features of *X*. Obviously, the size of this set can be indefinite, and not necessarily much smaller than that of *X*. Further, the quality of each processed feature can benefit from a large number of samples.

The deep convolutional neural network is a technique that follows the latter paradigm and exploits every feature. During its progress from one layer to another, it produces more features by combining and mixing the individual features; then, assesses their contribution, and finally feeds them back to the input in subsequent iterations. The price here is twofold. The design of the suitable convolutional deep network requires making several decisions including the selection of layer types and the way they are connected, from infinite possible choices. As there are currently no scientific rules that result in these decisions, most research rely on trial and error methods. The other challenge is tracing back the output features to their input origins. The hierarchies of transformations that are applied to the data creates a huge network of parametrized activations. Traversing the path from each deep layer feature to its associative ancestors is the first step in interpreting the model.

## 2. Materials and Methods

We have described the dataset and the data preparation process in the subsequent section, and then introduced our deep neural networks.

### 2.1. Datasets and Data Preparation

We retrieved the CNV data for six cancer types from the cBioPortal for Cancer Genomics [21]. The portal provides CNV calls that were generated from segmentation and marker files using genomic identification of significant targets in cancer algorithm, by setting four thresholds that divide the CNV spectrum into five regions/labels [22]:

−2: corresponds to the deletion of both copies
−1: corresponds to the deletion of one copy
0: corresponds to having exactly two copies (normal state)
1: corresponds to a low-level gain
2: corresponds to a high-level gain

The total number of the downloaded cancer samples was 3480, the breakdown of which is listed in Table 1. Each of the samples consists of CNV labels for 24,174 genetic cytobands. The dataset was transposed to have the features of each sample in a row.

**Figure 1:**
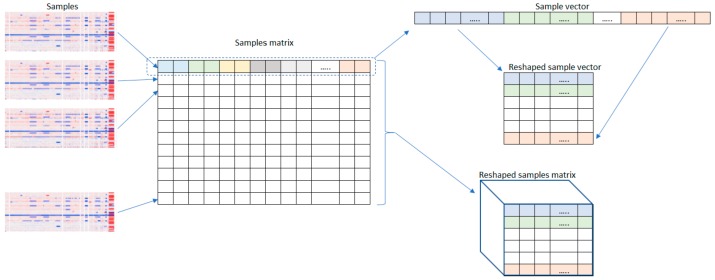
Data preparation process.

In this work, 2D-deep neural networks were utilized, which required the data to be in a rectangular form. Arranging the features in a 2D structure brings the initial features closer to each other and gives them a better chance to mix, with distant features, earlier in the process Initially, the vector values of each sample were scaled to the range [0, 255] and padded with zeros, which correspond to the absence of CNVs, so that the count of its elements was a perfect square (i.e., $156^{2}$); then, it was reshaped into a square matrix; and finally, resized by padding zeros to the size of (224 × 224). A channel depth of three was obtained by combining three copies of the developed matrix. Figure 1 depicts the steps in this process. At the end of this stage, our dataset can be described by the following two variables:

(1) $$Scaled\;CNV\;values:\;c\;\in \;{ℜ}^{M\times M\times S},$$

where: *M* = 224, the size of each dimension of the square matrix, *S* = 3,480, number of samples in the dataset.

Class labels:

(2) $$l\;\in \;\Sigma^{S},$$

where:$\;\Sigma =\left\{BRCA,\;COAD,\;GBM,\;KIRC,\;OV,\;UCEC\right\}.$

### 2.2. Convolutional Neural Network Layers

The building blocks in our model were neural network layers. We used eight types of layers that are commonly used in designing deep learning networks. This section explains the functions of these layers and demonstrates mathematically how they processed our data.

a. **Input layer:** This layer accepts a sequence of 2D (224 × 224) matrices and applies data normalization to each matrix. Each element of the sample matrix is given as:

(3) $$c_{ij}:\;i,\;j\in \left[1,M\right].$$

b. **Convolutional layer:** This is an essential part of each stage of the network and aims to extract patterns that are common in all training samples by applying a convolutional operation between a set of *k* sliding filters *f* (usually called a filter bank) and the output matrix of the previous layer. If the filter, $f_{k}\in {ℜ}^{M^{\prime }\times M^{\prime }}$; then, the result from this operation is given as:

(4) $$v_{ijk}=b_{ijk}+\sum_{d=1}^{D}f_{k}\cdot sub\left(c_{ijd},M^{\prime }\right),$$

where: *D* is the number of filters to be applied and the depth of the resulting matrix, $sub\left(c_{ijd},M^{\prime }\right)$ is a submatrix of *c*, with size $M^{\prime }\times M^{\prime }$ with $c_{ijd}$ as its center, · is the dot product between two matrices, $b_{ijk}$ is a bias value that helps the network to learn thresholds.

c. **Rectified linear unit layer:** It serves as an activation unit by ensuring that the values of its output are all positive. The output is given as:

(5) $$r_{ijk}=max\left(v_{ijk},0\right).$$

d. **Batch normalization layer:** It performs normalization on the convolution output of each stage, over a batch of samples.
e. **Pooling layer:** It divides its input into pooling regions and aggregates their information; hence, it reduces the dimensions of the features. In our model, we used a max-pooling layer that produced the maximum of each region of size $M^{\prime \prime }\times M^{\prime \prime }$. When used with stride t, the output matrix will be of size $M_{p}\times M_{p}$:

(6) $$M_{p}=\frac{M-M^{\prime \prime }}{t}+1.$$

Each of the output elements is given by the formula:

(7) $$\begin{array}{ll} p_{i^{\prime }j^{\prime }k}= & \max\left(r_{i^{\prime \prime }j^{\prime \prime }k}\right)\;:\;\\ & (i^{\prime }\times M^{\prime \prime }-t)<i^{\prime \prime }\leq (i^{\prime }\times M^{\prime \prime }+t)\;,\;\left(j^{\prime }\times M^{\prime \prime }-t)<j^{\prime \prime }\leq (j^{\prime }\times M^{\prime \prime }+t\right) \end{array}$$

f. **Fully connected layer:** It produces a vector, of which each element is calculated based on all activations resulting from the previous layer, by multiplying it by a matrix of weights $W_{fc}$ and adding a vector of bias offsets.

(8) $$fc_{i}=W_{fc}\times P+b_{fc}.$$

g. **Dropout layer:** This layer prevents the model from overfitting by randomly setting its input to zero according to the chosen probability.
h. **Softmax layer:** This layer maps its input to a normalized probability distribution over the output classes. The mapping uses the softmax function.

### 2.3. Convolutional Neural Network Architecture

One significant challenge in the design of a deep neural classifier is the manual selection of the optimal configuration of the network. Configuration refers to the selection of the depth of the layers, function of each layer, size and count of filters to be used in each layer, parameters for applying each filter, and the connections among layers. Unlike the filter weight values, which are learnt during the course of training, these parameters are required to be selected manually before the training begins.

**Figure 2:**
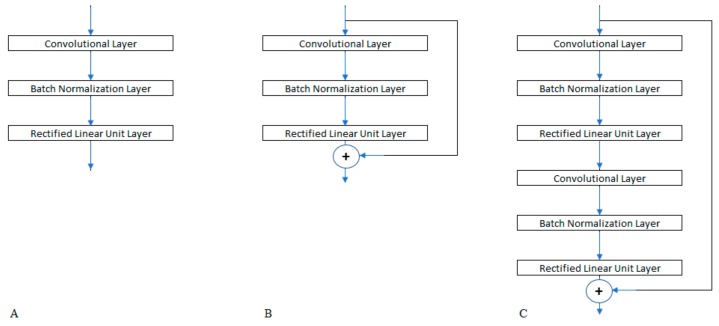
Network stage (**A**) without residual shortcut, (**B**) with residual shortcut for one stage, and (**C**) with residual shortcut for two stages.

To investigate the possible architectures for our classifying network, we defined a convolution stage that was created with three layers: a convolutional layer, a rectified linear unit layer, and a batch normalization layer. We examined the configurations that resulted from the combinations of ten design choices, which are subsequently summarized:

1. Positioning the batch normalization layer before or after the rectified linear unit in each stage.
2. Variations in the number of stages: 2, 3, 4, 5, 6, 7. We stopped at 7 as there was no indication of an enhancement in accuracy in our trials, while the parameter calculation penalty increased rapidly.
3. Variations in the convolution filter size: 3, 5, 7. Each stage may use a different size from other stages.
4. Variation in the initial number of filters in the first stage: 4, 8, 16, 32.
5. Determining if the number of filters in each stage is identical to, or double that of the previous stage.
6. Choosing the use of average or max feature extraction in the pooling layer.
7. Variations in the pool size (stride value) in the pooling layer: 2,4.
8. Variations in the dropout probability: 0.5, 0.6, 0.7, 0.8, 0.9
9. Determining the number of fully connected layers and the size of each. The combination of these two factors varies in the lists: (2048), (1024), (512), and (256) for a single fully connected layer, and (2048, 256), (1024, 16), and (512, 64) when two fully connected layers are used.
10. Using a residual shortcut link to sum the output of a stage to its input. There are three variations with respect to this factor, as illustrated in Figure 2: omitting the residual connection, having a residual connection for each stage, and connecting the output of each stage at an even order to the input of the previous stage.

By growing the network incrementally and combining these variations, we ran 142 different networks on the same dataset and observed two metrics: accuracy and number of learnable parameters. We present two techniques for cancer classification based on CNAs. The first is to design and train two deep neural networks. Moreover, the second is to transfer the learning of a deep network that is pre-trained in a different domain and fine-tune it to successfully classify our data.

#### 2.3.1. CNN6: A Shallow Convolutional Network

**Figure 3:**
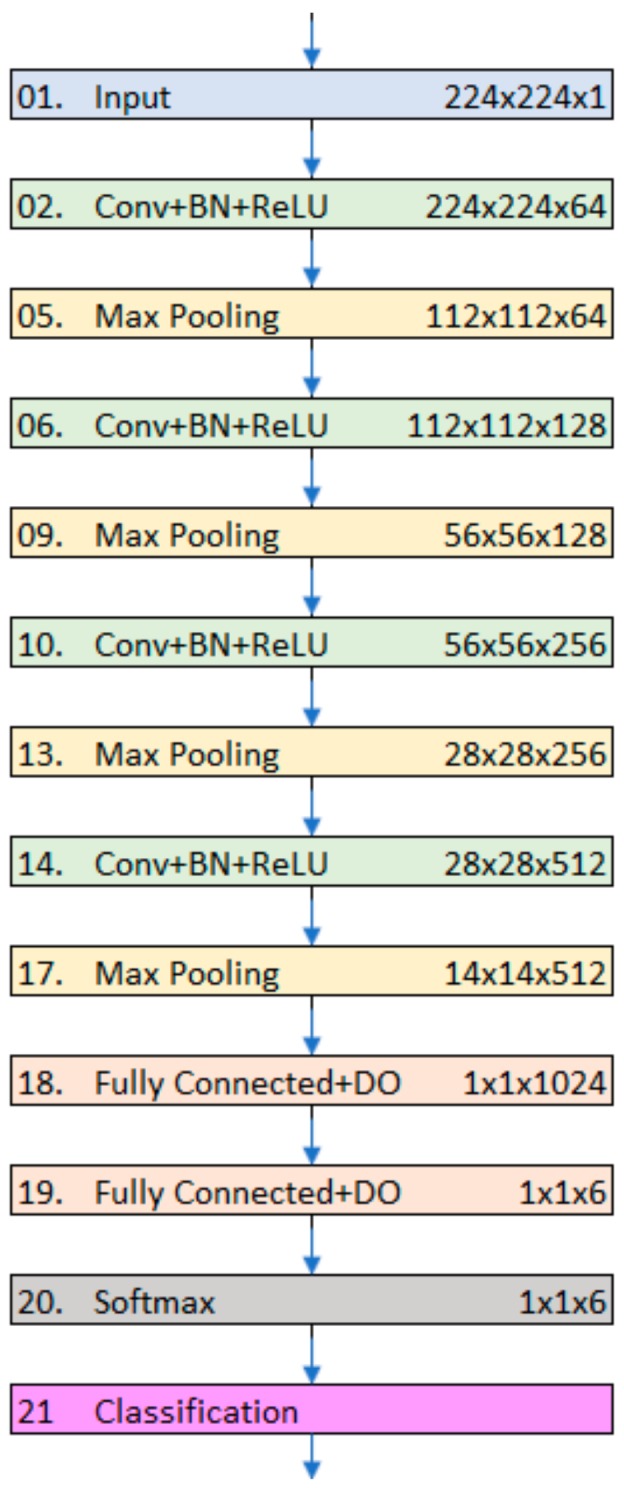
CNN6 architecture.

Our first convolutional neural network consisted of nineteen layers, of which only six were weighted. Figure 3 shows the architecture of the network. Through its learning process, the neural network applies a sequence of convolution, nonlinearity, and pooling operations to extract features with increasing levels of complexity.

Table 2 lists all the layers of our models along with their configurations. In the subsequent paragraphs, we present a brief description of the main layers of our model architecture.

#### 2.3.2. Residual CNN6 Network

He et al. [23] proposed the residual network as a solution to the degradation problem that arises with the increase in the network depth. The solution was to add a shortcut connection that bypasses one or more layers, to ensure that no deeper model produces higher training error than its shallower counterpart.

**Figure 4:**
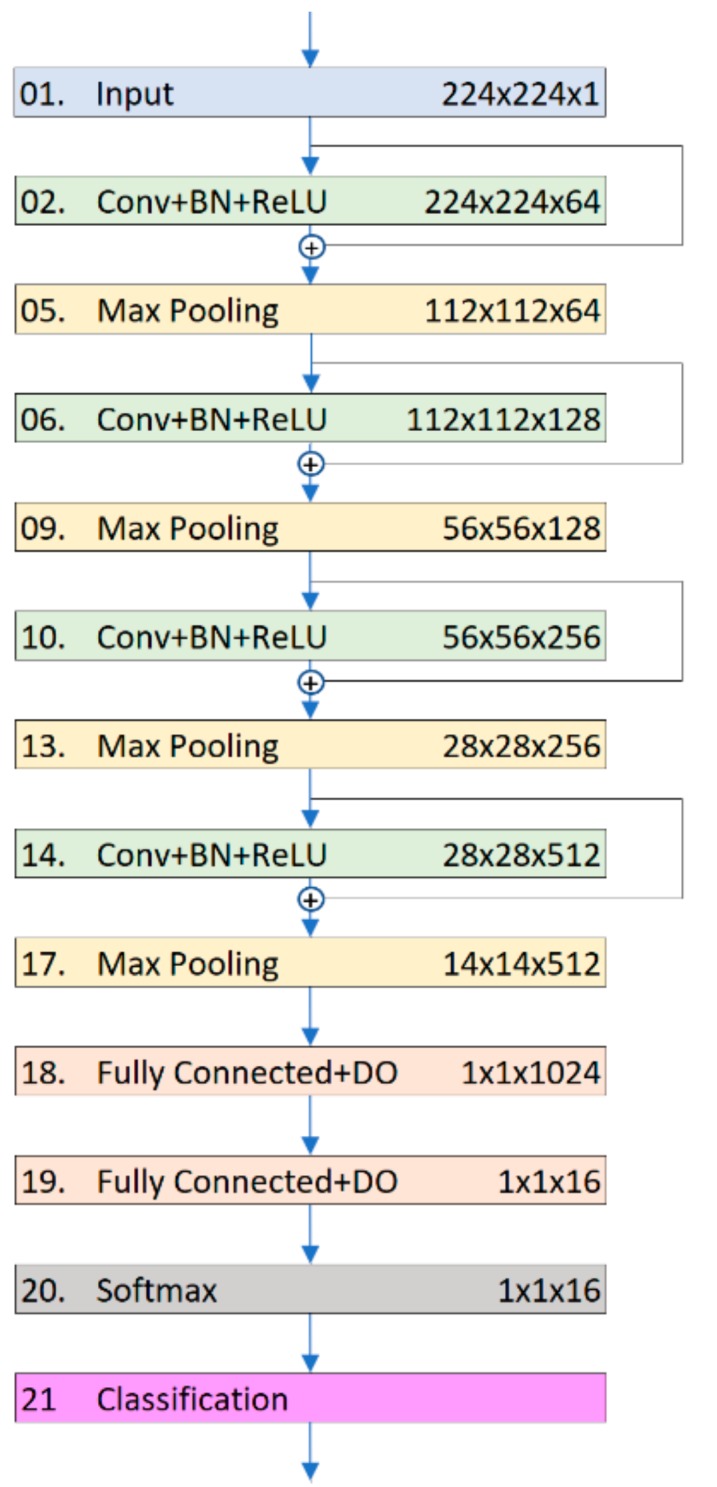
Residual CNN6 architecture.

We modified CNN6 to obtain the advantage of this feature, resulting in the residual CNN6 (ResCNN6). Figure 4 shows where the shortcuts are placed in the second model.

#### 2.3.3. Fine-Tuning A Pretrained VGG16 Network

VGG16 was introduced by the Visual Geometry Group at the University of Oxford. Here, sixteen indicates the depth of the weighted layers (thirteen convolutional and three fully connected layers) [24]. It was trained with more than a million images that belong to 1000 classes with over 370 K iterations to calibrate 138 M weight parameters. The image input size of the network is 224 × 224.

The transfer learning technique is performed by considering a pre-trained network as the starting point. The early layers of a CNN usually learn from low-level features and can be extracted to be reused in other applications. However, the later layers learn domain specific features and have to be replaced and tailored to fit the nature of the new dataset. Retraining the network with the new dataset (called fine-tuning) is much faster and easier than training a network that is built from scratch.

## 3. Results

We ran our experiments using the three deep models introduced in Section 2.2, on the dataset described in Section 2.1. The decision to choose this dataset was partially considered to enable an unbiased comparison to the Dagging classifier [15], which was evaluated against the same data.

### 3.1. Data Division

The dataset was split into 80% for training and 20% for testing (i.e., the classifier will not see testing samples until the training process is over). Twenty percentage of the training set was used for in-process cross validation following two different techniques: (1) holdout (2) 10-fold. In every dividing operation, a check was considered to ensure all classes were represented in the subsets proportional to the original dataset.

### 3.2. Deep Network Training Options

The deep models, CNN6 and ResCNN6, were trained, and the deep models and VGG16 were fine-tuned using the following setup:

- A training period spanned 50 epochs, each epoch contained 104 iterations, for a total of 5200 iterations.
- Adaptive learning rates started at an initial rate of 0.1% and dropped by a factor of 10 after every 10 epochs.
- A validation was conducted every 50 iterations.

After each run, the accuracy of predicting the test group and the corresponding confusion matrix were recorded.

## 4. Discussion

**Figure 5:**
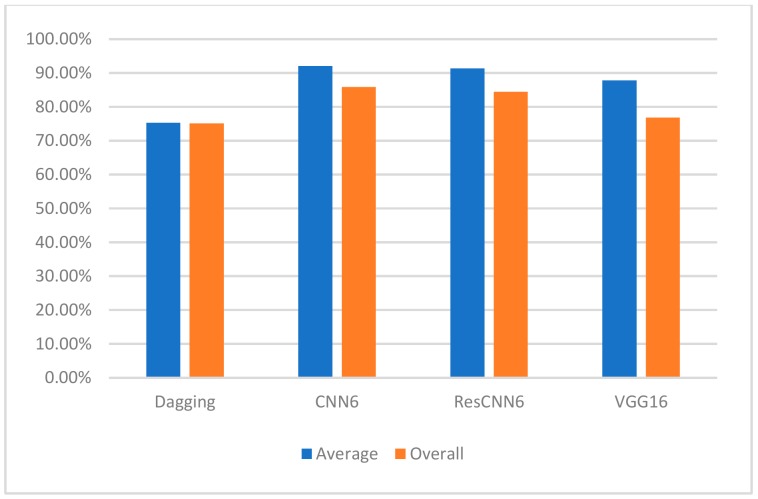
Comparison of the average and overall accuracies of the different classifiers.

We used three measures—accuracy, specificity, and sensitivity—to evaluate the performance of our three classifiers and the Dagging method. The reported overall accuracies were 84.4%, 85.9%, and 76.9% using CNN6, ResCNN6, and VGG16, respectively, which prove a considerable improvement when compared to the 75.1% accuracy achieved by Dagging. Figure 5 compares the average and overall accuracies of the dadding algorithm and the three convolutional classifiers.

**Figure 6:**
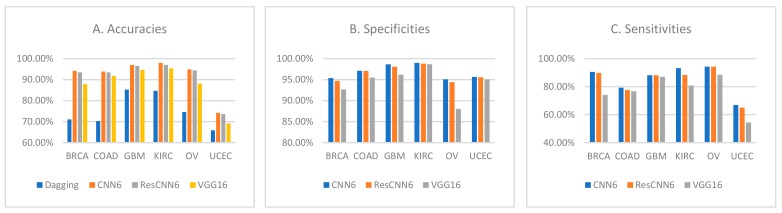
Comparison of the classifiers’ per-class (**A**) accuracies, (**B**) specificities, and (**C**) sensitivities.

The results demonstrated higher specificities for CNN6 and ResCNN6 when compared to VGG16. Finally, CNN6 achieved the highest sensitivity values for each class, followed by ResCNN6, and VGG16. We have summarized our findings in Figure 6.

**Figure 7:**
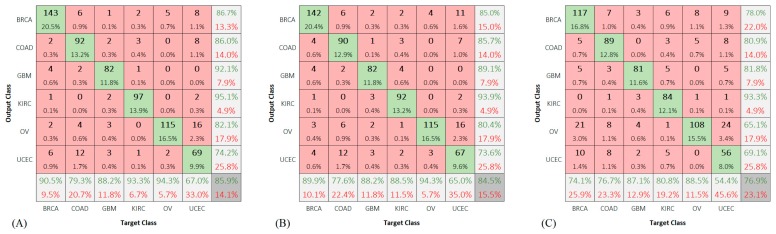
Confusion matrix for running: (**A**) CNN6, (**B**) ResCNN6, and (**C**) VGG16, on the test group.

We also included the confusion matrices for class predictions of the test group using the deep networks (Figure 7).

A high error rate was observed while predicting the UCEC samples. The reported accuracies for this class were 65.9%, 75.8%, 67.0, and 61.5% using Dagging, CNN6, ResCNN6, and VGG16, respectively.

We considered the subsequent assumptions for the classes OV and UCEC: (1) they have some features in common, and (2) the number of their corresponding samples is not enough to learn their features and tell them apart from each other. Although each of KIRC and OV classes has less samples than UCEC, they seem to possess distinguishable features.

**Figure 8:**
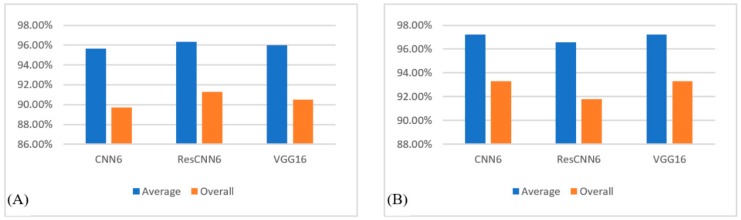
Comparison of average and overall accuracies for running the deep networks on five-class samples: (**A**) by omitting uterine corpus endometrial carcinoma samples and (**B**) by omitting ovarian serous carcinoma samples.

To test our first assumption, we repeated the training of the three deep networks using five classes twice, once by dropping the OV cases and the other by dropping the UCEC cases. Figure 8 shows the results of these experiments.

**Figure 9:**
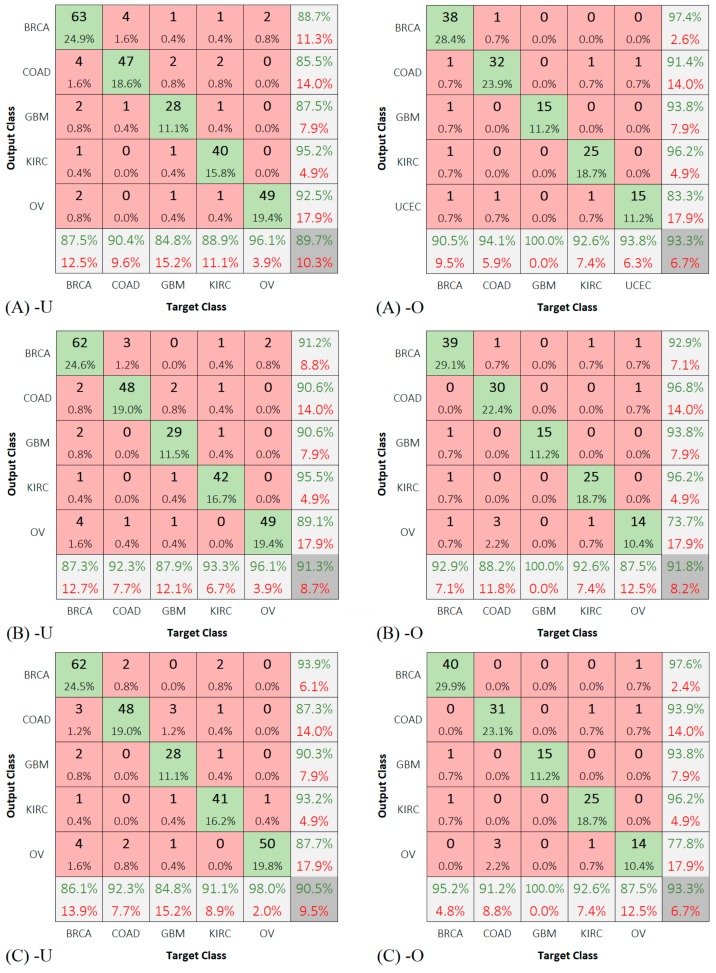
Confusion matrix obtained from running of (**A**) CNN6, (**B**) ResCNN6, and (**C**) VGG16, on the test group of five classes (-U: no UCEC and -O: no OV).

Figure 9 shows the confusion matrices for the predictions of the test groups of five classes only.

**Figure 10:**
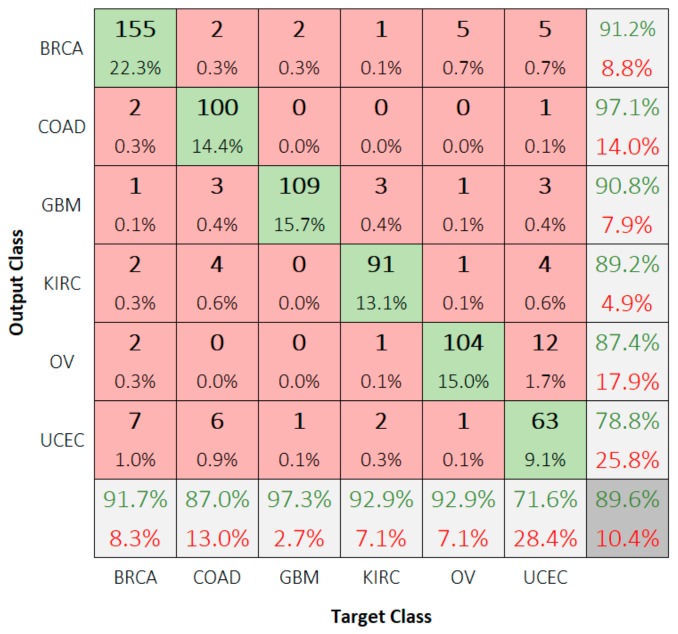
Confusion matrix for classifying the test dataset after training CNN6 using balanced oversampled training dataset.

The results show that the classes OV and UCEC demonstrate a significant gain when the samples of each are present separately. To test our second assumption, we trained our models on more samples of UCEC and OV classes. One common technique to achieve this was oversampling of the training set [25]. By repeating all observations of all classes until the number of samples of each class were equal to those of the majority class, we obtained a balanced dataset. However, precaution must be taken to avoid overfitting. Running the experiment again on the resulting dataset demonstrated an improvement in the accuracy. Figure 10 illustrates an overall accuracy of 89.6% and UCEC class-specific accuracy of 78.8% using CNN6.

## 5. Conclusions

In this paper, we presented three deep learning methods. The first consisted of a six-layer convolutional neural network while the second appended shortcut connections to the first method to form a residual version. The third technique involved the transfer learning of an accurate image classifier, VGG16, which was modified and fine-tuned to work with our data. The data that contained the samples of six cancer types were scaled and reshaped to fit the input layers of the classifiers.

Our experiments demonstrated significantly higher accuracies when compared to the state-of-the-art methods for solving this problem domain. The residual network was proven to be the most accurate amongst the attempted techniques.

Furthermore, we observed a reduction in the accuracy while predicting one of the tested classes. We hypothesized that the UCEC and OV share certain key features that misled the classifier. To test our hypothesis, we repeated the experiments twice, excluding one of these classes in each experiment. The results confirmed an improvement in the accuracy in both trials.

## 6. Future Work

Our work can be extended in three directions. The first is to investigate the CNV-based classification further, both by including other cancer types and by optimizing the deep networks. The second is to consider integrating non-CNV features for the samples we used. We expect to correct the classification errors by supplying other dimensions of genetic information. The third is to build a model that infers the contributions of the low-level features to their corresponding classes based on the high-level parameters of the deep network.

## Figures and Tables

**Table 1:** Number of samples and their percentages of each of the six cancer types.

Cancer Type	Coded As	Samples	Percentage
Breast invasive carcinoma	BRCA	847	24.34%
Colon adenocarcinoma/Rectum adenocarcinoma	COAD	575	16.52%
Glioblastoma multiforme	GBM	563	16.18%
Kidney renal clear cell carcinoma	KIRC	490	14.08%
Ovarian serous cystadenocarcinoma	OV	562	16.15%
Uterine corpus endometrioid carcinoma	UCEC	443	12.73%

**Table 2:** Layers of CNN6 and their configurations.

Layers	Configuration	Output Size
Input layer	-	224 × 224 × 3
Convolution 1 + BN ^1^ + ReLU ^2^	64 filters, 3 × 3 window	224 × 224 × 64
Max pooling 1	2 × 2 region, stride = 2	112 × 112 × 64
Convolution 2 + BN + ReLU	128 filters, 3 × 3 window	112 × 112 × 128
Max pooling 2	2 × 2 region, stride = 2	56 × 56 × 128
Convolution 3 + BN + ReLU	256 filters, 3 × 3 window	56 × 56 × 256
Max pooling 3	2 × 2 region, stride = 2	28 × 28 × 256
Convolution 4 + BN + ReLU	512 filters, 3 × 3 window	28 × 28 × 512
Max pooling 4	2 × 2 region, stride = 2	14 × 14 × 512
Fully connected + DO ^3^	Drop probability 50%	1 × 1 × 1024
Fully connected + DO	Drop probability 50%	1 × 1 × 6
Softmax + Classification	-	

^1^ Batch Normalization layer; ^2^ Rectified Linear Unit layer; ^3^ Drop Out layer.
